# Current Insights into Collagen Type I

**DOI:** 10.3390/polym13162642

**Published:** 2021-08-09

**Authors:** Ruth Naomi, Pauzi Muhd Ridzuan, Hasnah Bahari

**Affiliations:** 1 Department of Human Anatomy, Universiti Putra Malaysia, Serdang 43400, Malaysia; ruthmanuel2104@gmail.com; 2 Estika Research Centre, Kuala Terengganu 21080, Malaysia; drpmridzuan@gmail.com

**Keywords:** collagen type I, extraction, physicochemical, extracellular matrix, regenerative medicine

## Abstract

Collagen type I (Col-I) is unique due to its high biocompatibility in human tissue. Despite its availability from various sources, Col-I naturally mimics the extracellular matrix (ECM) and generally makes up the larger protein component (90%) in vasculature, skin, tendon bone, and other tissue. The acceptable physicochemical properties of native Col-I further enhance the incorporation of Col-I in various fields, including pharmaceutical, cosmeceutical, regenerative medicine, and clinical. This review aims to discuss Col-I, covering the structure, various sources of availability, native collagen synthesis, current extraction methods, physicochemical characteristics, applications in various fields, and biomarkers. The review is intended to provide specific information on Col-I currently available, going back five years. This is expected to provide a helping hand for researchers who are concerned about any development on collagen-based products particularly for therapeutic fields.

## 1. Introduction

Collagen (Col) is a triple-helix structure that can initiate and maintain the interaction between cells and matrix. To date, 28 different types of Col have been identified [1]. Col type I (Col-I) is the most common type of protein and makes up 90% of the human body. It is commonly found in the skin, bones, capsule of organs, tendons, cornea, and fascia [2,3] except in cartilaginous tissues [4]. Most commonly, Col-I is also known as fibril-forming Col [5], which is also most widely used in tissue engineering as a biomaterial due to its abundance [6]. Mineralized Col-I resembles the extracellular matrix of human skin due to its similar composition. Col-I is usually designated to the 3D Col matrix using a freeze-drying process, where the pore size is tuneable by temperature and velocity of the freeze dryer [4]. As it is the main structural constituent, the Col fibril influences the mechanical strength of tissues and organs [6]. Native Col comprises the polypeptide chain which, is known as the alpha molecule (α). This denotation indicates the type of Col. For instance, Col-I is made up of a gene known as COL1A1 and COL1A2, known as α1 and α2 [1,2,7]. COL1A1 consists of (G→T) Sp1-binding polymorphism, particularly in the first intron. This functions mainly to accelerate the affinity of the transcription process of Sp1, therefore promoting the expression of gene [7].

Col-I can either appear as a homotrimer or heterotrimer depending on the variants [1]. The homotrimers of Col-I play an essential role in wound healing [8]. Heterotrimers are the dominant isoform of Col-I, while homotrimers of triple-α helix chain usually occur in fibrotic lesion, fetal tissue, and some tumors. This usually happens due to the nature of the homotrimers, which are highly resistant to collagenase, leading to a hindered cleavage process. Approximately more than 1000 amino acids make up Col-I, and its length can reach up to 300 nm with a width of 1 to 5 nm. Col-I comprises three domains, namely N telopeptide, C telopeptide, and a central domain. In this, the central domains accommodate up to 95% of the molecule structure of Col-I [9]. Generally, Col-I is said to be highly resistant toward proteolytic degradation. However, certain proteases such as MMP-1, also known as interstitial collagenase and MMP-8, which is also known as neutrophil collagenase, still have the capacity to degrade Col-I [10].

### 1.1. Structure of Collagen Type I

**Figure 1:**
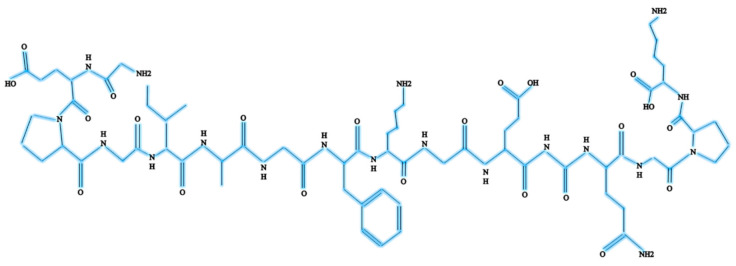
Chemical structure of Col-I.

Col-I is known as a triple-helical domain due to the structure of Col-I in the form of G-X-Y. In this, Glycine (G) is a constant amino acid while x and y can be any amino acids. Usually, x and y are occupied by proline and hydroxyproline [8,10]. All these three units will constantly appear as repeated units. Glycine at the third position is essential for ensuring the formation of the helical structure [11]. At a later stage, this will be packed into a different dimension of hexagonal and quasi-hexagonal shapes to form fibrillar Col. Therefore, Col is commonly found as an elongated fibril [12]. Figure 1 shows the chemical structure of Col-I.

**Figure 2:**
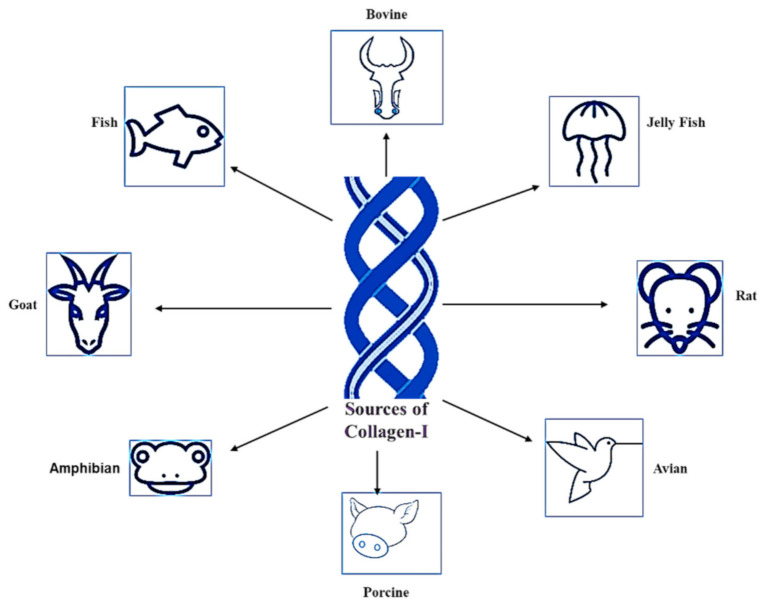
Sources of Col-I.

The Col-I molecular mass is 300,000 Dalton, which is equivalent to 300,000 g/mol with the molecular formula C_2_H_5_NOC_5_H_9_NOC_5_H_10_NO_2_ [12]. Post-translational enzymatic hydroxylation on specific proline and lysine occurs depending on the type of Col. The stability of the triple-helix structure depends on the formation of the intramolecular hydrogen bonds with the presence of 4-hydroxyproline [13]. Fibril-forming Col, especially Col-I, demonstrates essential characteristics to assemble in high molecular order with diameters between 25 and 400 nm. This remarkable property has been used to characterize the banding pattern of Col fibrils using transmission electron microscopy. A banding pattern with a periodicity of 70 nm is commonly found depending on the arrangement of Col monomers [14]. Meanwhile, pH, ionic strength, and temperature influence the formation of the fibrillogenic of Col-I. Molecules present in Col-I, under certain conditions, have the ability to form macroscopic fibers, microscopic fibrils, and fibril bundles instantaneously through self-assembly [15]. On top of that, Col-I is made up of 2 respective genes, namely COL1A1 located at the 17q21.3-q22 (long arm), and COL1A2 located at the 7q21.3-22.1 in the long arm of the chromosome. Col-I is distributed ubiquitously in both healthy and diseased tissue [11]. The size of the intron of these two genes varies greatly. The gene size of COL1A1 can rise to 18 kb while for COL1A2 to 38kb [8]. Figure 2 shows the molecular structure of Col-I.

### 1.2. Sources of Collagen Type I 

Silvipriya et al. (2015) described the available sources of Col-I from various species [16]. This includes mammalians, amphibians, fish, marines, birds, and human recombinant collagens [16,17]. However, bovine, porcine skin, and bones are classified as the traditional sources for Col-I, which have been documented since the early 1950s [18]. The sources of Col-I are summarized in Figure 2. However, the selection of Col-I usage is widely influenced by the source. Inevitably, each source has its own pros and cons. 

For instance, Col-I from bovines are easily accessible due to its wide range of availability, high biocompatibility, biodegradability, and low immunogenicity [19]. Unfortunately, bovine derived from Col-I lacks essential amino acids. Therefore, it is termed as an incomplete protein source for Col-I [20]. Moreover, approximately 3% of the human population has proven allergic to bovine Col-I. The outbreak of diseases such as bovine spongiform encephalopathy, or mad cow disease, and transmissible spongiform encephalopathies makes the use of bovine derived from Col-I risky [16]. Moreover, Cheng et al. (2017) stated that Col-I is a major component in jelly fish, specifically in the species *Rhopilema esculentum*. Those Col-I have been proven to be rich in minerals and protein [21], and cost-effective [22]. Rittié (2017) described that Col-I formed from rat tail is readily available and easy to extract. However, a high level of purification protocol must be handled, as it is easy to contaminate it [23].

On the other hand, avian influenza has restricted the choice of using avian for Col-I extraction [24]. Nonetheless, porcine Col-I is almost similar to human Col [16] and it is the cheapest source of Col-I [25]. However, it poses a high risk of zoonosis transmission, contamination, and broad constraints due to religious practices [16]. Ovine-derived Col-I is absent from any risk of transmission of disease and cultural insensitivity (halal certified) [25]. Col-I extracted from fish exhibits a high solubility rate in diluted acid compared to the Col-I extracted from mammalian and avian species. They even show a high degree of similar structure in both the α1 and α2 chain compared to other Col-I-derived sources. However, Col-I from fish sources exhibits a low level of denaturation temperature [19].

## 2. Collagen Type I Synthesis

**Figure 3:**
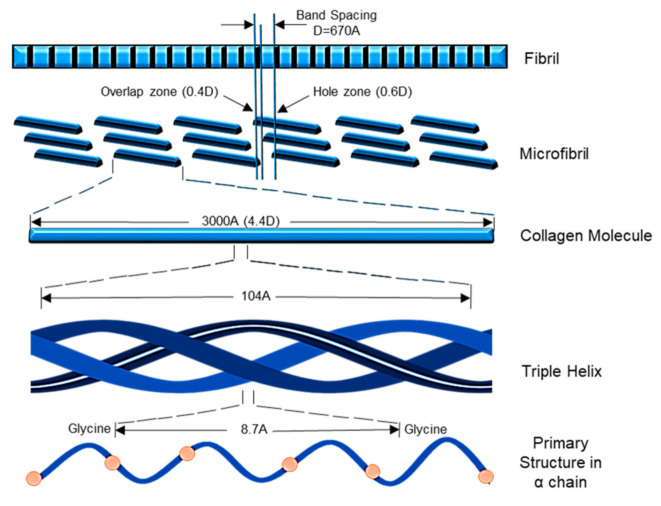
Synthesize of Col-I.

The synthesis of Col-I is a complex process. This includes a wide range of transcription of genes in the nucleus of cells to the formation of heterotrimer structures and fibrils (Figure 2). The synthesis comprises of transcription and translation, post-translation, formation of the triple helix, and secretion of Col-I. This usually occurs in the general route starting with the removal of propeptide preceded by the lysyl-crosslinks formation [9,11,15,16,26]. The synthesis process of Col-I has been summarized in Figure 3.

### 2.1. Transcription

The type of cell, growth factors, and cytokines greatly influence the regulation process of transcription in Col-I. Most collagen usually comprises complex patterns of exons and introns. The exons usually range from 3 to 117. In this scenario, mRNAs of fibrillar Col are encoded by more than 50 exons. This is the main reason for the detection of different types of mRNA species in many cases. This can also be due to the presence of various initiation sites or the alternative splicing of exons [8]. In addition to the splicing, pre-mRNA undergoes capping at the 5′ end, and polyadenylation at the 3′ end. In this phase, the genes encoded for pro α1 and pro α2 chains are transcribed in the nucleus [16,17].

### 2.2. Translation

In this phase, the mature mRNA will be transported into the cytoplasm before being translated at the rough endoplasmic reticulum. In this stage, the mRNA bounded to the ribosome will be translated into pre-procollagen. Upon translation, this is known as pre-pro-polypeptide chain [27]. This, with the assistance of signal recognition domain, will protrude into the rough endoplasmic lumen for the post-translation process. At the end of this process, this phase will be recognized by the corresponding receptors [8].

### 2.3. Intracellular Post-Translational Modifications

The post-translational modification process is initiated upon the removal of the signal peptide. This is removed by signal peptidase. Prolyl 3, 4, and lysyl hydroxylase will catalyze the residues of proline and lysine hydrolyzation. Catalyzation by all these 3 enzymes is possible with the assistance of co-factors such as oxoglutarate, ascorbate, molecular oxygen, and ferrous ions [8]. In proline residues, up to 50% is comprised of the hydroxyl group. This exists at position 4. The extent of the prolyl-hydroxylation is known as species dependence. The 4-hydroxyproline is vital for the formation of the intramolecular bond that exists between collagens. Glucosyl and galactosyl residues are transferred to the hydroxyl groups of hydroxylysine. Hydroxylysyl galactosyltransferase and galactosylhydroxylysyl-glucosyltransferase enzymes will then catalyze the transferred residues [28].

The assembly of the three α chains into the monomers of collagen trimeric is greatly influenced by C-propeptides [29]. Meanwhile, N-linked carbohydrates and intra-chain disulfide bonds stabilize the C-propeptide globular structure. The stabilizers will be then be added to the complex oligosaccharyl transferase enzyme [30]. The alignment of the C-terminal domains of the three α-chains determines the formation of the triple-helical structure. This is followed by the initiation of the formation of the triple helix [31].

### 2.4. Collagen Type I Triple-Helix Formation

In this phase, the disulfide bond will be formed by the cysteine, which is a part of the C-propeptide. During Col secretion, this bond will disintegrate together with the removal of the propeptides. This makes for a solid cohesion intracellularly, therefore avoiding any sliding of α-chains against one another. The triple-helical structure then propagates from the C- to the N-terminus in a zipper-like form. For a triple helix of type I procollagen to form takes an average of 14 min to fold. Following the triple-helical folding, hsp47 will attach to the procollagen. This bonding to G-X-Y repeats in the Y region with Arg. The triple helix consequently provides stability and prevents premature procollagen aggregation. Nonetheless, hsp47 separates the endoplasmic reticulum after the procollagen shift to the Golgi apparatus modification. In particular, for Col-I, the trimmer will comprise of pro α_2_(I) or α1(I)_3_ and pro α1(I)_2_ [15].

### 2.5. Collagen Type I Secretion

The helical structure will be then packed into a secretory vesicle in the Golgi apparatus. This happens after the assembly of procollagen has been achieved. Upon packaging, this will be released into the extracellular space. The trimers of procollagen will be processed based on the type of collagen, following secretion. The C-propeptides and N-propeptides will be cleaved into two specific proteases. This is now known as the N-proteinase and the procollagen C-proteinase [32]. 

## 3. Extraction and Purification of Collagen Type I

### 3.1. Acidic/Salting-Out Extraction Method

Col-I extraction can be achieved through an acidic/salting-out procedure [33]. This method is commonly used to extract Col-I mainly from soft tissue such as skin. Initially, from the non-collagenous region, the skin will be removed. The removed skin is then defatted using sodium chloride (NaCl) and hydroxymethyl aminomethane hydrochloride (Tris-HCI) [34]. It will be then sliced into pieces and will be soaked in 0.5 mol/L of acetic acid with a 1:50 ratio of solid to solution [35] at a temperature of 4 °C for about 24 h [36]. The extraction was then filtered under the same conditions. The supernatant was removed. This was followed by salting-out by adding 1.5 M of NaCI for 24 h. After 24 h, the precipitate was collected and centrifuged at 10,000× *g* for about 15 min. The resultant was subsequently re-dissolved in acetic acid with a concentration of 0.5 M [35]. The step of salting-out and solubilization was repeated 3 times. The precipitate resultant was then dialyzed using cold water using a dialysis tube with a cut-off of molecular weight of 8000 Da, then freeze-dried [36] and stored below −20 °C [35]. The principle of the salting-out method is to neutralize the salt ions carried by the NaCl to the collagen molecule surface charge. It eventually reduces the electrostatic force that exists between the collagen molecules, which assists in collagen precipitation [37].

### 3.2. Alkali Extraction Method

Alkali is another method used in Col-I extraction, and this method is used to extract Col-I from various sources, especially leather waste. In this method, the Col-I can be successfully extracted due to the strong capacity of alkaline to hydrolyze Col fibers [37]. Briefly, the skin was soaked in the alkaline solution containing sodium hydroxide solution (NaOH) with a pH of 12 [38]. This was then followed by placing it in a rotary evaporator at a speed of scale 6 at a temperature of 60 °C using a 10 L flask. The incubation took an hour for an extraction concentration of 125 gL^−1^ to be obtained and filtered out using a double-layered gauze. A step of centrifugation was performed for 10 min at 8000× *g* followed by freeze-drying and storage until further use [39]. Thus, the alkali-based extraction method is dependent on the principle of destroying all the amino acid-containing sulfhydryl and hydroxyl to ensure success rate in a full precipitation step [37].

### 3.3. Acid Extraction Method

Col-I extraction is based on an acid approach initiated with the disruption of inter- and intra-covalent bonds of the particular sample. Generally, raw material such as porcine or fish skin has been soaked in 0.5 M acetic acid solution until it is fully swelled, for up to 72 h, eventually removing the non-collagenous protein. The precipitation will be then collected through a centrifugation step, and treated with salt solution (NaCI) to separate the supernatant. The supernatant was removed, and clotted precipitate was separated. The procedure is followed by the dialyzed process for 2 days, with distilled water changed every 12 h to obtain the purified Col-I [34]. This approach involves the basic destruction of the amino acids in the Col-I structure that will release Col-1 from the collagenous protein [27,40].

### 3.4. Enzymatic Extraction Method

The enzymatic method is used to obtain biologically active Col-I protein without damaging the amino acids [37]. In the enzymatic protocol, the raw material will be soaked with an acetic acid solution that has been mixed with enzymes. The most commonly used enzymes include flavourzyme, alcalase, pepsin [34], papain, or trypsase [37]. Before being filtered out, this mixture will be continuously under the controlled temperature of 4 °C, for up to 48 h. This enzymatic process is continued by filtration to obtain a precipitate followed by the dialyzed steps for 2 days with the interchange of distilled water every 12 h to obtain the purified Col-I [34].

## 4. Potential 3-Dimensional (3D) Design of Collagen Type I

Col-I can be fabricated into different forms of 3D designs, such as films, sponges, membranes, nanofibers, sheets, beads, meshes, hydrogel, gel, microspheres, and composite scaffold [41,42,43]. Col film is primarily used as a barrier in tissue engineering. Relative to ophthalmological barriers, films with a thickness ranging from 0.1 mm to 0.5 mm can be cast from Col solutions through air drying. Films produced from biodegradable materials such as telopeptide-free reconstituted Col provides a smooth release of encapsulated drugs that can be categorized as an additional benefit. The mounted films have effective sterilization and are malleable after hydration, without losing their mechanical force [44].

Col sponges are usually prepared from lyophilization or the freeze-drying process. Through this process, the pore size can be modified. This enables the capacity of the Col sponge to absorb a massive amount of tissue exudates, maintain a moisture microenvironment and easy adherence to the wounded region, and provide a barrier against mechanical stress and microbial infection. Col sponge implantation of burn wounds causes rapid skin regeneration due to an intensive penetration of sponge neutrophils. Col sponges are incredibly beneficial in wound healing, as their moist consistency helps soft tissue to be perforated and provides a basis for a new growth of tissues. Col-I sponges can accelerate wound healing by fostering the deposition of Col fibers parallel to the Col-I sponge. This in turn increases the tensile strength of open wounds, therefore contributing to the rapid healing mechanism [45]. At the same time, Col-I sponges are ideal for the deliverance of short-term antibiotics at the wounded region, with the absence of adverse effects. Interestingly, this sponge can be absorbed into the native tissue over a certain period of time [46].

Col membranes are widely used for dural seals, wound dressing, and guided tissue regeneration (GTR), particularly in the dental field [47]. Usually, single films are linked together through gentle pressure to form the Col membrane. Since platelet-derived growth factor (PDGF) released from the Col membrane can maintain a constant pace, up to 100 h, it is scientifically proven to enhance the healing mechanism [48]. Furthermore, biodegradable Col membranes are viable fibroblasts and support tissue growth. A hybrid of the Col membrane, such as the atelocollagen matrix, further enhances the long-term survival of fibroblasts by stimulating attachment and proliferation [49].

Similarly, Col-I nanofiber is considered to be superior due to its out-ranging benefits. Native Col-I nanofibers are produced through electrospinning, and they are mechanically weak. Therefore, they are always integrated with other synthetic polymers to optimize the mechanical strength without altering the biological properties. Electrospun collagen nanofiber mesh has a high surface-to-volume ratio, a tuneable diameter and porosity, and good biological activity to control cell structure and shape of the tissues [50]. Nevertheless, electrospun Col nanofibers can imitate ECM structure; biological cues also have a major effect on cell behavior. Due to this, Col-I nanofibers have been screened for the regeneration of tissues and organs [51].

By contrast, Col-I sheets have been proven to be effective in treating clean chronic wounds. Col-I sheets not only promote early healing, but are also cost-effective, decreasing the rate of secondary infection and the need for analgesics. This can be due to the structure of the Col sheets, which are thin and easy to integrate at the wound site [52,53]. Nonetheless, Col-I in the form of beads can be used for the expansion of cells, direct delivery, and reabsorbable implantation [54]. Conversely, Col-I meshes are used to restore weak tissues, specifically for hernias, oral or pelvic floor [55].

Furthermore, Col-I, being a hydrophilic substance, can be easy tuned into a hydrogel. In this form, Col-I can hold >90% water [56]. Due to its large uniform surface area, it is suitable to be used as a drug-delivery system. Commonly, Col-I hydrogel is better combined with either natural synthetic polymers to further enhance its biocompatibility and mechanical properties [57]. Col-I, which is modified in the form of a gel, can be used as an injectable material. In this scenario, the injection of Col-I gel into the spine system enables exons to arise from the interface and then grow into the implanted Col gel within a short duration [58]. Apart from this, Col-I modulated into the microsphere is known to be the perfect biomaterial for the deliverance and regeneration of neural and neural progenitor cells [59].

## 5. Physicochemical Properties of Collagen Type I 

The physiochemical properties of Col-I greatly differ based on source of extraction. The related parameters that are extensively evaluated for the physical characterization of Col-I 3D stability consist of the assessment of mechanical strength, thermal stability, microporous study, swelling ratio, water vapor transmission rate, and surface characteristics. Meanwhile, X-ray photoelectron spectroscopy (XPS), Fourier transform infrared (FTIR), energy-dispersive X-ray (EDX), and X-ray diffraction (XRD) were studied for chemical characterization. 

### 5.1. Physical Characteristics

#### 5.1.1. Mechanical Strength

The stability of Col-I is influenced by mechanical strength, thermal stability, porosity, and biodegradation. It is essential to develop a bioscaffold that is adequately stable and does not affect biological functions [60]. Moreover, structure stability helps to cue cell behavior within the three-dimensional structure. Several factors contribute to mechanical strength including the design of the three-dimensional scaffold [61,62] and crosslinking intervention [34,35]. Different sources of Col-I derivative do not show a significant difference in tensile strength. However, ovine-derived Col-I was superior compared to bovine, porcine, and rat derivative. Ovine Col-I shows ultimate stress of 15.08 ± 2.89 kPa with a strain percentage of 50.74 ± 4.02%. Porcine derivative Col-I exhibits ultimate stress of 13.91 ± 3.11 kPa with a strain percentage of 47.15 ± 6.20% while for bovine it was 12.33 ± 2.37 kPa and 34.87 ± 5.83%, respectively [63]. Meanwhile, native Col-I extracted from rat tail with a length of 3.5 ± 0.5 µm (persistence length) shows a Young’s modulus of 11 MPa to 95 MPa with a contour length of 6.9 ± 2.2 µm [64].

#### 5.1.2. Thermal Stability Denaturing Temperature

Col-I from different sources, such as species and tissue type, has a different denaturing temperature (Td) [65]. This affects the 3D bioscaffold stability and causes handling difficulties, especially in fish and marine collagens [40]. A previous study by Subhan et al. (2015) reported that the Col-I denaturing temperature is approximately at 29 °C, 40.8 °C, and 37 °C for jelly fish, mammals, and porcine, respectively. Thermal stability has a direct correlation with denaturing temperature [66]. Col-I stability is greatly influenced by the presence of glycine and hydroxyproline content, and this greatly differs among different species. For instance, Col-I derived from the marine species Chondrosia reniformis consists of 18.9% of glycine and 40% hydroxyproline. This indicates that the marine-derived Col-I is less stable due to a low composition of amino acid compared to mammals [67]. However, with current advanced technology, the desired stability of fish collagen bioscaffold can be achieved via crosslinking [66]. This further improves the denaturing temperature of fish collagen, resembling mammalian collagen for future use in clinical applications.

#### 5.1.3. Porosity and Pore Size

A 3D scaffold is essential for providing a better microenvironment with suitable pore sizes and porosity for cell survival and fate inside layer structure. This environment should mimic the nature of the tissue extracellular matrix (ECM) that varies considerably depending on tissue type. This allows the cells to behave and interact according to mechanical cues provided by the designated bioscaffold [68]. Generally, a smart bioscaffold should consist of the porous structure with highly interconnected pores where it allows nutrient and oxygen diffusion and the removal of biological waste, and creates a 3D environment in the scaffold for the assembly of cells and differentiation. Moreover, perfectly interconnected pores make cell seeding, growth, proliferation, and formation of new tissue easier by providing sufficient space in the scaffold [69]. For instance, a porosity of more than 90% is required to support osteoprogenitor cells, and this is an ideal scaffold for bones [70].

A well-interconnected pore structure ensures homogenous cell distribution on the scaffold [71]. A pore size within the range 150–250 µm is an ideal value for cell distribution, which smooths cell delivery onto the Col-I scaffold [69]. However, optimum pore size differs depending on the type of cells. This is because a pore size of >80 µm is ideal for the ingrowth of fibrogenic growth, while <20 µm is necessary for chondrogenic growth. Meanwhile, pore size differs based on Col-I derivative. In this scenario, the average mean of pore size recorded for ovine-, bovine-, and porcine-derived Col-I scaffolds are 73.05 ± 10.79 µm, 85.84 ± 9.51 µm, and 87.32 ± 10.69 µm, respectively [63].

#### 5.1.4. Swelling Ratio

Swelling is an important element not only for determining the efficiency of the Col-I scaffold but also for the water adsorption capacity of the scaffold. The swelling ratio depends on the degree of crosslinking, presence of hydrophilic groups, pore size, and interconnection among the pores [72]. The swelling ratio differs based on the source of the Col-I derivative. For instance, ovine-derived Col-I has twice the resistance towards collagenase degradation compared to bovine and porcine-derived Col-I. The estimated swelling ratio of ovine, bovine, and porcine-derived Col-I are 2500%, 2750%, and 2700%, respectively [63]. The swelling ratio of pure Col-I is approximately about 650% due to the presence of hydrophilic groups [73]. In tissue engineering, swelling ratio favors the lesion exudate environment to ensure the desired wet surroundings at the injury site. A range between 24.05 g/g and 45.65 g/g is desirable for exudate adsorption in burns. Thus, it is classified as a perfect bioscaffold attachment for the injury site, which later expedites tissue regeneration [74].

#### 5.1.5. Water Vapor Transmission Rate

Water Vapor Transmission Rate (WVTR) indicates moisture permeability of a Col-I scaffold. This is an essential component in wound healing, as WVTR ensures adequate moist surface surrounds the wound. An optimum range of WVTR correlates with good drainage of exudation at the injury site. WVTR differs based on Col-I derivative. For example, Col-I derived from fish scales and porcine demonstrated 952.6 ± 55.5 g/m^2^/day and 1090.9 ± 77.1 g/m^2^/day [75]. A low level of WVTR will cause the accumulation of exudates while an extreme level of WVTR water evaporates at the injury site, leading to a state of dehydration. An optimum range of WVTR within the range of 2028.3  ±  237.8 g/m^2^/day is essential to maintain a moist environment, as well as to enhance the normal healing phase [76].

#### 5.1.6. Surface Characterization

The cell–scaffold surface interaction plays a main role in cell attachment, proliferation, and tissue regeneration [77]. It is an essential factor that governs cell response at adherence stage. Cells are intended to attach to the hydrophilic surface rather than the hydrophobic one. This is because in a hydrophilic environment, high amounts of water molecules and nutrients can be retained. This will be used by cells that are being seeded on the scaffold. Therefore, there will be an increased level of cell spreading, attachment, and proliferation onto the scaffold [78]. The presence of Col-I provides suitable wettability (hydrophilicity) as the collagen molecule consists of hydrocarbon chains and hydrophilic functional groups [79].

Contact-angle measurement shows the perfect wettability surface for a Col-I surface with a presence of better hydrophilicity. Briefly, the contact angle will be measured at different time frames to obtain the average value. The decrease in contact angle was notable, with interval time of up to 12 s. In this scenario, a high degree of contact angle >90% indicates a low level of hydrophilic and wettability property of the Col-I bioscaffold [80]. The water contact angle (WCA) on acid-soluble Col (ASC) and pepsin-soluble Col (PSC) from the Col-I derivative of red stingray shows significantly different results. The study showed that WCA for ASC was 100.3°± 2.31° with a contact angle of 30.67° ± 1.89° while for PSC it was 94.96° ± 0.59° with a contact angle of 42.00° ± 1.14°. The results indicate that both are hydrophobic (>90°) due to the presence of amino acid and pepsin residues in ASC and PSC, respectively [81].

Similarly, atomic force microscopy (AFM) provides topological information at the nanoscale level [82]. The AFM study reveals that Col-I is present with homogenous fibril arrangements in the D-band. In hydrophilic states, Col-I forms a strong interaction with other polymers with a physiological property of D-periodicity at ~67 nm. AFM shows that photodegradation is essential for modifying the surface roughness of Col-I. In this case, low UV radiation is suggested to decrease the surface roughness, as imposing UV radiation has an effect on cell behavior in later phases [83].

### 5.2. Chemical Characteristics

The structural and chemical modification of Col-I to develop 3D scaffolds could change the chemical structure of Col-I. Thus, its chemical characterization will help to determine the existence of a basic molecule for Col-I. Common techniques that are used, including X-ray photoelectron spectroscopy (XPS), Fourier Transform Infrared (FTIR), Energy-Dispersive X-ray (EDX), and X-ray Diffraction (XRD), are briefly reviewed in this part. 

#### 5.2.1. X-ray Photoelectron Spectroscopy (XPS)

X-ray photoelectron spectroscopy (XPS) is a common technique to quantify the elemental composition, including chemical state and electronic state, of elements that exist within biomaterials. Moreover, XPS uses a high radiation of synchrotron, which has the capacity to detect surface implants and characterize different surfaces in Col-I [84].The maximum evaluation of XPS samples must be within 20 cm^2^, with a height less than 25 mm. Samples analyzed in XPS show a ≈0.1 atomic% as nominal sensitivity with an elemental sensitivity, which may differ by as much as ≈100. Meanwhile, for the assessment of chemical components, a sample size larger than ≈10 μm will be convenient [85].

For instance, XPS study was performed on covalently immobilized Col-I on biomimetic surfaces by Scarano and co-researchers in 2019 in the presence of a thin film of oxide layer to cover the titanium surface. The result obtained for the control measure (titanium surface) shows a composition of 49.2% oxygen, 31.6% carbon, and 0.4% nitrogen on titanium coating. On the other hand, a surface coated with titanium and Col-I exhibited a composition of 23.8% oxygen, 59.3% carbon, and 14.3% nitrogen [84]. The XPS study in this experiment reveals the surface content of the proteinaceous layer, which proves the titanium surface is successfully coated by collagen molecules.

#### 5.2.2. Fourier Transform Infrared (FTIR)

Infrared (IR) spectroscopy has been used to detect the vibration characteristics possessed by specific functional groups in a material. The functional group will absorb IR radiation at a specific wavenumber (cm^−1^) range, where it represents a chemical bonding vibrating at a specific frequency [86]. Col-I has several functional groups that encompass Amide I, II, and III. Furthermore, these amide groups are detected at a range of peak intensity between 1450 cm^−1^ and 1235 cm^−1^ and commonly indicate the helical structure of Col-I. These amide groups are not able to differentiate the type of Col. However, Riaz et al. (2018) reported that the presence of Amide A at the higher peak intensity of 3350 cm^−1^ can be attributed to Col-I [87]. Peak intensity of 1632 cm^−1^ indicates the higher-order arrangement of collagen structure that refers to β-sheet and triple-helix structure [33].

#### 5.2.3. Energy-Dispersive X-ray (EDX)

Elemental analysis materials are important for determining the biomimetic and biocompatibility for biomaterials development. Energy-dispersive X-ray is an analytical evaluation to measure the major element composition of a material. The major elements in Col-I are oxygen, nitrogen, and carbon, with a higher percentage of oxygen followed by nitrogen and carbon. This indicates that the Col-I resembles the normal composition of elements found in the human body [88]. The same ratio of elemental content in Col-I to the tissue indicates biocompatibility, which explains its native properties have been preserved, even though, with certain post-intervention, that allows the cells to migrate and grow throughout the bioscaffold [89].

#### 5.2.4. X-ray Diffraction (XRD)

The atomic structure of Col-I can be acquired via X-ray diffraction technique. XRD assists in determining the materials in the crystalline or amorphous phase. Electromagnetic waves influence the X-ray diffraction data obtained. Stronger signals appear in the crystal samples more easily, due to the presence of crystallinity structure. A crystalline sample is periodic, and comprises cells in three independent directions. This appears in random orientations—amorphous, powder, or liquid state. More importantly, the orientation of Col-I fibrils is disrupted in those states, causing the loss of orientational information. Col-I demonstrates the amorphous phase instead of crystallinity [33]. Thus, Col-I has a random arrangement of atomic structure with inappropriate diffraction and low captured signal. The XRD of collagen generally consists of 2 clear peaks. The first peak is usually sharper than the second peak [90]. This serves as the main reason for the appearance of Col-I snippet-ordered structure, particularly in the amorphous state. Nonetheless, XRD has proved that Col-I derivatives from different sources are closer to the amorphous phase than the crystallinity.

#### 5.2.5. Power of Hydrogen (PH)

The pH value of Col-I determines its potential application in any field of interest. It greatly differs regarding the source of derivative and method of extraction. For Col, the isoelectric point of Col-I represents the pH value, and this is usually measured in pKa. Native Col-I has a lower isoelectric point, which is approximately at a pH of 7.2, suggesting a low intermolecular interaction compared to other types of Col, with a high amphiphilic charge density on the surface [91]. The average pKa value for amine groups in Col-I is approximately 9.3, whereas for carboxylic acid it was 4.1 when the Col-I was suspended in an aqueous solution with a pH of 6.6 [92].

## 6. Biomarkers for Col-I

Abnormalities or mutations in the Col-I gene can lead to the occurrence of certain diseases. This includes Ehlers–Danlos syndrome classical type, Ehlers–Danlos syndrome type VIIA, osteogenesis imperfecta types I–IV, idiopathic osteoporosis, and Caffey disease [93,94,95,96,97]. These can be diagnosed using biomarkers. Biomarkers for Col-I can be classified into two categories, namely degradation and synthesis biomarkers. Degradation biomarkers are further divided into Col-I neoepitope (C1M), C-terminal telopeptide of Col-I (CTX-I), and Col-I-derived crosslinked carboxy-terminal telopeptide (ICTP). Furthermore, synthesis biomarkers are broken down into carboxy-terminal propeptides of pro Col-I (PICP) and amino-terminal propeptides of pro Col-I (PINP) [98].

## 7. Application of Collagen Type I 

Despite the different sources of derivative, Col-I is highly biocompatible and exhibits low immunogenicity due to its structure being similar to humans. Nonetheless, Col-I is widely available, either commercially or extracted from a variety of sources such as animals, humans, insects, bacteria, and plants. Therefore, Col-I is widely incorporated in a wide range of fields. This includes tissue engineering, medical devices, the pharmaceutical industry, and the biomedical field. 

### 7.1. Tissue Engineering

Native Col-I is well known for its biocompatibility in human tissue, regardless of the source. This property makes Col-I a gold standard for the incorporation of Col-I in tissue engineering [18]. The tuneable characteristics of porous Col-I [99] further enhance the wide integration of Col-I as the main choice of biomaterial in tissue engineering. Col-I has been widely used as a regenerative for nerve deficits [100], regeneration of bone [101], chondrogenic differentiation [102], vascular grafts [103], and skin substitutes [104].

Klein et al. (2016) accessed the outcome of Col-I for digital nerve repair in the forearm region. They noticed that about 0.5 mm of Col-I scaffold was enough to give a satisfactory outcome. Through their study, they witnessed >64.8% mean recovery rate in motor nerves and the disability of arm, shoulder, and hand score was in the minimum range, which was 17.0 [100]. Similarly, Samadian et al. (2019) noticed that Schwann cells showed rapid proliferation in an implanted Col-I scaffold. Furthermore, in vivo study showed a functional index of sciatic −22.13  ±  3.00 on the 60th day post-Col-I scaffold implantation. Histological analysis shows uniformity in fiber arrangement with an intact myelin sheath formation [105]. Conversely, Zhang et al., (2018) studied the effect of integrating Col-I for bone regeneration. Col-I, being the main organic components in bone, with some modification, serves as a template for the mineralization to progress. They found that the combination of Col-I and hydroxyapatite perfectly resemble the hypoxic environment in the osteogenic niche. The implanted scaffold showed that it has the ability to recruit host cells and accelerate mineralization, therefore enhancing bone regeneration [106].

On the other hand, Kumar et al. (2013) noticed that a hybrid of elastin and Col-I provided a resilient matrix as well as being thrombo-resistant when the designed scaffold was implanted as a vascular graft. At the same time, the scaffold exhibited stability over long durations, thus modulating thromboresistance [107]. At the same time, there is less possibility for immune response stimulation, which further proves that Col-I is a primary choice for biomaterial of vascular graft [108].

### 7.2. Medical Devices

Modified Col-I is widely used as a bio-ink when producing bioprinting due to its high cytocompatibility with human cells. This is mainly because the Col-I has the ability to self-organize into fibrils under neutral-condition D-skin substitutes. Artificial human hepatocytes, direct neural cell printing, and cornea-like cell-laden structure are some of the examples of Col-I-based bioprinted materials [109].

In relation to the use of Col-I in medical devices, 3D bioprinting has gained the attention of researchers. In this scenario, scientists have successfully developed patterned tissues, implantable scaffolds, micro-physiological devices, perfusable vascular networks, etc. In this case, Col-I has served as the primary choice of biomaterial because the composition and microstructure are always tunable according to need. At the same time, Col-I as a bio-ink has been speculated as a highly compatible biomaterial, since it plays a critical role in ECM, can enhance the uniformity in structural and tissue arrangement, can increase mechanical strength, and can act as a template for tissue adhesion and signaling molecules [110].

By contrast, 3D skin substitute, a bioproduct of bioprinting developed from Col-I, provides new therapy for chronic skin loss. This is because it is developed with the capacity to replicate the growth factor and inflammatory agents such as cytokines and enzymes. This is to ensure optimum restoration and regeneration of skin is achieved. Currently, Apligarft^®^, Integra™, Epicel^®^, Epidex™, Myskin™, ReCell^®^, Dermagraft^®^, and OrCel^®^ are some of choices available for 3D skin substitutes [111].

### 7.3. The Pharmaceutical Industry 

The use of Col-I in the food industry is boundless. Col-I has the potential to be incorporated as a wound dressing, drug-delivery device, injectable dispersion, microparticle, and Col shield in ophthalmology sponges. This is possible because Col-I has been approved as a gold standard for its biocompatibility, which further enables the use of Col-I for cell culture systems. In particular, in the pharmaceutical industry, Col-I has high potential for use due to its natural characteristics such as high cell affinity, biodegradability, and weak antigenicity [112].

In the pharmaceutical field, Col-I is modified into nanospheres, nanoparticles, or microspheres to be used as a device for drug delivery. In this form, they have been proved to be effective in penetrating systemic circulation with the aid of Col-I. This is possible because the integration of Col-I has made it able to cover large surface areas due to its small size, high absorption ability, and the capacity to form a colloidal solution [113]. In optometry, Col-I has been known to be a gold standard as a Col shield. Despite being a bandage for short period, it allows sufficient exchange of oxygen molecules to ensure continuous metabolism in the cornea. Bearing this in mind, and due to its proven positive outcome, there are a few FDA-approved Col-I shields. These include Biocora^®^, Proshield^®^, MediLenso^®^, Irvine^®^, and Chiron [114].

Col-I in the form of injectable material has also gained much attention [115]. Usually, this will be initially developed as a gel and will be converted into fiber-injectable suspensions or non-fibrillar viscous solutions to ease application. This is possible because upon soaking in any biological solution, Col-I can maintain its integrity. Specifically, for corneal application, specified biological fluid can be combined with drugs and administered. Interestingly, upon administration, Col-I is still able to maintain its native state as a liquid solution, which, later on, will transform into gel independently [114].

### 7.4. Cosmeceutical

Col-I is also extensively used in the cosmeceutical industry as an anti-ageing product, dermal filler, and for skin repair. Since Col-I is a natural anti-oxidant, it can be used to maintain hydration level in skin, elasticity, minimizer for wrinkles, and substitute for photo-damaged Col fibers. As such, more and more anti-wrinkling, UV protection, and anti-ageing creams and gels are being introduced. This mainly correlates to Col’s ability in cosmetic formulations to eliminate trans-epidermal water leakage, and protection from corrosive chemicals [116].

Apart from this, Col-I has been widely used as a Botulinum toxin type A (injection), soft-tissue filler, chemical peel, and for laser hair removal. This was certified as a non-invasive procedure in the United States in 2014. For instance, Zyderm^®^ and Zyplast^®^ have been accredited as the most commonly used dermal fillers in the US to date. Furthermore, porcine-derived Col-I has been proven to reduce acne scars and wrinkles over a short period with the absence of adverse side effects. Bearing this in mind, porcine-derived Col-I is considered to be the safest in cosmetic applications in comparison to other sources of Col-I [117].

### 7.5. The Food Industry

The use of Col-I in the food industry has no boundaries. Col-I has been used as food additive, edible film and coating, supplement, drink, and carrier. As food additive, Col can maintain the rheological properties of preserved foods such as sausages. Furthermore, it serves as an emulsifier in acidic foods, which aids the increment in creamy substances. Furthermore, Col improved the physicochemical properties of threadfin bream and sardine surimi. Col-I can also be used as edible coating for fillet, meat paste, boneless ham, etc. This reduces the shrinkage while preserving the juiciness as well as acting as a barrier against fat oxidation. Therefore, Col-I edible films may replace plastic coatings as they resist fat oxidation, discoloration, and microbial development, and retain sensory qualities [24].

## 8. Recent Advances of Collagen Type I

**Figure 4:**
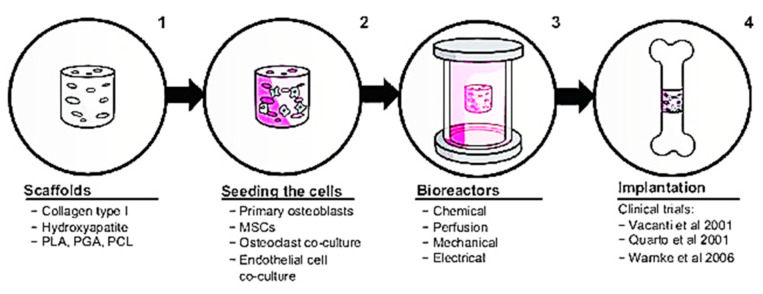
Col-I in bone repair. Figure is reused from Rupani et al. (2012) [121]. Used under the Creative Commons Attribution—Non Commercial (unported, v3.0) License. http://creativecommons.org/licenses/by-nc/3.0/ accessed on 8 May 2012.

Numerous biological characteristics of Col-I have caught the attention of researchers to further explore the possible application of Col-I in the biomedical industry. This section explains recent advances of Col-I in the research field. Regarding this claim, a recent discovery shows that Col-I can control the nucleation and development of bone mineral crystals as well as the formation of sacrificial bone. This is achieved as Col-I forms a bond between mineralized Col fibrils that exist in the bone to further enhance the toughness of the bone. Additionally, by removing osteopontin, a non-collagenous protein in the bone matrix, Col-I fibrils can be reorganized in the bone tissue [116]. Furthermore, by introducing synthetic polymers, such as polyvinyl alcohol, composite membranes containing Col-I and bone particles, the modulus and tensile strength of membranes can be increased by up to 10 wt. % [118]. Three-dimensional printing has been used to produce bone regeneration scaffolds made of composite calcium phosphate and Col [119]. This model depicts a mimetic hybrid osteocalcin–fibronectin fusion protein with a Col-binding domain and fibrillar collagen matrices. Surprisingly, over the course of a month, this hybrid biomimetic matrix demonstrated excellent structural stability, rapid anchoring of mesenchymal stem cells, high cell proliferative potential, and increased in vivo bone growth in calvarial defects [120]. Col-I in bone repair involves a four-step in vitro tissue-engineering method, as shown in Figure 4. Initially, a biomaterial scaffold will be used as a flat sheet to simulate skin or a bundle of fibers to substitute a 3D cylinder to be transplanted into the bone. After that, stem cells will be used to bioactivate the construct. The construct will be cultivated in a bioreactor after the cells have been seeded, replicating at least one feature of the in vivo environment, such as tensile strength or mechanical stress. The final stage is to implant the construct and, if possible, observe its behavior ex vivo.

Additionally, many skin substitutes are also Col-I derivatives that are available for clinical trials. For instance, a recent invention, Advanced Wound Bioengineered Alternative Tissue, is a porous silicone membrane attached to a continuous 3D nylon framework containing non-crosslinked porcine Col-I peptides. This product is still under trial for its effectiveness, vulnerability towards infection, and fluid entrapment in the wounded region. Other skin substitutes, Apligraf^TM^ and OrCel^TM^, are formed with fibroblasts implanted into a bovine Col-I matrix, and with keratinocytes cultivated at the air–liquid interface. It is used to graft onto partial-thickness wounds and provides a suitable matrix for host cell migration. Matriderm^®^ is another product that consists of bovine Col-I and elastin. It is used for dermal regeneration, where the ECM is laid down by fibroblasts and the Matriderm^®^ reabsorbs around the wounded region during the healing process [122].

## 9. Current and Future Prospective

The unique properties exhibited by Col-I make it suitable to be modified and incorporated in various fields specifically for therapeutic use. Although different sources of Col-I describe different properties, generally, all are worth being manipulated due to their high biocompatibility and cell affinity compared to other types of Col. This serves as another gold standard for the implementation of Col-I in various fields. Meanwhile, a limited number of functional groups for crosslinking and mechanical resistance toward vascular extracellular matrix (ECM) hinders Col-I from being inculcated into clinical settings worldwide. It is still a great challenge to reproduce and optimize collagen in a safe, low-cost, and reliable way to be used in the therapeutic field. Therefore, it is still a novelty to explore Col-I synthesis from various natural sources, and further exploration is much needed to cater to the current limitations. Therefore, this might meet the demand of current medical needs, given advancing globalization. In future, more research must be done to ensure Col-I mimics the ECM biologically and chemically, despite different sources of extraction, for a better performance.

## Data Availability

The data presented in this study are available on request from the corresponding author.
